# Phytochemical Composition and Antioxidant Activity of Various Extracts of Fibre Hemp (*Cannabis sativa* L.) Cultivated in Lithuania

**DOI:** 10.3390/molecules28134928

**Published:** 2023-06-22

**Authors:** Asta Judžentienė, Rasa Garjonytė, Jurga Būdienė

**Affiliations:** Center for Physical Sciences and Technology, Department of Organic Chemistry, Sauletekio Avenue 3, LT-10257 Vilnius, Lithuania; rasa.garjonyte@ftmc.lt (R.G.); jurga.budiene@ftmc.lt (J.B.)

**Keywords:** *Cannabis sativa* L. ssp. *sativa*, *Cannabaceae*, essential oils, extracts, gas chromatography, mass spectrometry, high-performance liquid chromatography, diode array detector, time-of-flight mass spectrometry, amperometry, hydrogen peroxide scavenging, antioxidant, prooxidant activity

## Abstract

The phytochemistry of fibre hemp (*Cannabis sativa* L., cv. Futura 75 and Felina 32) cultivated in Lithuania was investigated. The soil characteristics (conductivity, pH and major elements) of the cultivation field were determined. The chemical composition of hemp extracts and essential oils (EOs) from different plant parts was determined by the HPLC/DAD/TOF and GC/MS techniques. Among the major constituents, *β*-caryophyllene (≤46.64%) and its oxide (≤14.53%), *α*-pinene (≤20.25%) or *α*-humulene (≤11.48) were determined in EOs. Cannabidiol (CBD) was a predominant compound (≤64.56%) among the volatile constituents of the methanolic extracts of hemp leaves and inflorescences. Appreciable quantities of 2-monolinolein (11.31%), methyl eicosatetraenoate (9.70%) and *γ*-sitosterol (8.99%) were detected in hemp seed extracts. The octadecenyl ester of hexadecenoic acid (≤31.27%), friedelan-3-one (≤21.49%), dihydrobenzofuran (≤17.07%) and *γ*-sitosterol (14.03%) were major constituents of the methanolic extracts of hemp roots, collected during various growth stages. The CBD quantity was the highest in hemp flower extracts in pentane (32.73%). The amounts of cannabidiolic acid (CBDA) were up to 24.21% in hemp leaf extracts. The total content of tetrahydrocannabinol (THC) isomers was the highest in hemp flower pentane extracts (≤22.43%). The total phenolic content (TPC) varied from 187.9 to 924.7 (average means, mg/L of gallic acid equivalent (GAE)) in aqueous unshelled hemp seed and flower extracts, respectively. The TPC was determined to be up to 321.0 (mg/L GAE) in root extracts. The antioxidant activity (AA) of hemp extracts and Eos was tested by the spectrophotometric DPPH^●^ scavenging activity method. The highest AA was recorded for hemp leaf EOs (from 15.034 to 35.036 mmol/L, TROLOX equivalent). In the case of roots, the highest AA (1.556 mmol/L, TROLOX) was found in the extracts of roots collected at the seed maturation stage. The electrochemical (cyclic and square wave voltammetry) assays correlated with the TPC. The hydrogen-peroxide-scavenging activity of extracts was independent of the TPC.

## 1. Introduction

The genus *Cannabis* has a long and complicated history of botanical classification, and to date, its taxonomy remains controversial [1,2,3]. Nowadays, it is mostly considered that the genus has only one species, *Cannabis sativa* L., separated into two subspecies: ssp. *sativa* (containing a low amount of the psychoactive constituent Δ^9^-tetra-hydrocannabinol (THC), usually less than 0.3% in the dry weight of the upper third of flowering plants) and ssp. *indica*, so-called drug (medical) cannabis (with high amounts of THC). The two subspecies can be separated into wild and domesticated varieties: under ssp. *sativa*, var. *sativa* is domesticated and var. *spontanea* is wild, and under ssp. *indica*, var. *indica* is domesticated and var. *kafiristanica* is wild [1,2,3,4]. Fibre hemp *C. sativa* ssp. *Sativa*, having relatively high heterogeneity, is comprised of many cultivars and varieties.

Hemp may have grown naturally on the territory of Lithuania around 5500 years ago, only later becoming a cultivated plant [5]. The plant, together with millet (*Panicum* sp.) and wheat (*Triticum* sp.), was undoubtedly one of the first crops cultivated on the Lithuanian territory in the middle-Neolithic period (3500–3000 BC). Several types of hemp grains and their fibre ropes have been found in the Šventoji settlements (Lithuania) from the Narva culture (Mesolithic period) [5]. 

Today, hemp is a well-known plant due to its widespread cultivation throughout the world. It is an eco-friendly crop that complements a sustainable growth system and can be grown under a huge variety of agro-ecological conditions, even without herbicides, fungicides or pesticides [6,7]. One hectare of hemp sequesters 9 to 15 tons of CO_2_, similar to the amount sequestered by a young forest, but it only takes five months to grow [7]. Hemp is a crop grown across all of Europe. In recent years, the area dedicated to hemp cultivation has increased significantly (by 75%) in the EU from 19,970 ha in 2015 to 34,960 ha in 2019 [7], and according to the European Industrial Hemp Association (EIHA), the hemp cultivation area in Europe was 50,081 ha in 2018 [8]. In the EU, in the same period, the production of hemp increased by 62.4% (from 94,120 to 152,820 tons) [7]. France is the largest producer, accounting for more than 70% of EU production, followed by the Netherlands (10%) and Austria (4%) [7]. With 9182 ha of hemp fields (in 2019), Lithuania is among the ten largest industrial hemp growers in Europe [9]. The following hemp varieties are mostly cultivated in Lithuania: Beniko, Wojko and Bialobrzeskie (acquired from the Institute of Natural Fibers and Medicinal Plants in Poznań, Poland), and several cultivars, including Epsilon 68, Felina 32, Santhica 27, Fedora 17 and Futura 75 (purchased from French growers), and USO 31 (of Ukrainian origin) [9,10]. Hemp was banned due to its visual resemblance to medical cannabis in many countries (including Lithuania), and only since 2013 has fibre hemp been legal in Lithuania.

The possibilities of producing already known and creating new products from hemp are very high, and the application of these products is wide in many industries (e.g., pharmaceuticals, perfumery, food, bio-energy, agriculture, etc.). Industrial hemp comprises fibre and oilseed hemp [11,12]. Strong, durable and antimicrobial hemp fibre is produced from the stems, which have long been used to make textiles, paper, ropes, etc. [11,12,13,14,15,16,17,18]. Hemp fibre can replace wood, plastic, metal, and various other types of fabrics and building materials [6,15,16,17]. Trees need several decades to mature, while hemp grows in a hundred days and produces more cellulose of better quality that does not require complex chemical processing [9,10]. Hemp stalks and chaff are also used in the horticulture, livestock, construction and other industries [16]. Hemp seeds, containing a high quantity of proteins, polyphenols and fatty oils, have commonly been claimed as a nutritionally complete food source [16,18,19,20,21]. Seeds and products made from hemp seeds are attractive to consumers looking for organic food, as well as those with coeliac disease and lactose intolerance [18]. Hemp seed oil consisting of 80–90% of unsaturated fatty acids is a rich and balanced nutritional source with antioxidant properties that is able to replace classic animal fat [20,22,23,24]. Hemp roots are a valuable resource for agricultural (mainly as compost) and industrial applications (for paper production) [25]. The roots of the cannabis plant have a long history of medical use for treating mainly inflammation and pain [26].

Hemp leaves and flowering tops are rich in terpenoids, flavonoids, phenolic acids, cannabinoids, vitamins and minerals [9,10,11,12,13,14,15,16,17,18,19,20,21,22,23,25,26,27,28,29,30,31,32,33,34,35,36,37,38,39,40,41,42,43,44,45,46,47,48,49,50,51,52,53,54,55,56,57,58,59,60]. Owing to the presence of valuable bioactive compounds, various fibre hemp extracts can exhibit antimicrobial [28,29,30,31,32,33,34,48,49], anti-inflammatory [35,48], antifatigue [36], antioxidant [20,41,42,43,44,45,46,47,48,49], antiproliferative [46], cytotoxic [49], insecticidal/pesticidal [50,51] and allelopathic [52] properties.

Antioxidant activity (AA) is related mostly to polyphenolic compounds. Usually, AA is evaluated spectroscopically in vitro, employing the abilities of polyphenols to scavenge free radicals 1,1-diphenyl-2-dipicrylhydrazyl (DPPH^●^), 2,2-azino-bis(3-ethylbenzothiazoline-6-sulfonic acid (ABTS^●+^) or reactive oxygen species (ROS), or to form complexes with transition metals [61,62]. Electrochemical techniques, such as cyclic voltammetry, differential pulse or square wave voltammetry, have been applied for the evaluation of the antioxidant properties of various objects, such as beverages, plant extracts or individual polyphenols [63,64,65,66,67,68]. The electrochemical approach is based on the physicochemical properties of compounds and can, therefore, be considered as a direct test for antioxidant properties.

Most of the studies on essential oil (EO), cannabinoid and polyphenol production have been carried out for the strain selection of drug (medical)-type *C. sativa*. On the contrary, there is still a lack of studies on the phytochemistry and AA of different aerial parts and roots of fibre-type *C. sativa* cultivated in various countries. It should be mentioned that phytochemicals in cannabis roots and stems are not well characterized [26,35,45,69,70].

Herein, we investigated the:

(i) Main soil characteristics (conductivity, pH and major elements) of fibre hemp cultivation habitat;
(ii) Chemical composition of cultivated hemp (*C. sativa* ssp. *sativa*) EOs obtained from inflorescences, leaves (during various growth stages) and stems;
(iii) Chemical composition of volatile organic compounds (VOCs) in *C. sativa* extracts of flowers, leaves, unshelled seeds and roots (collected at different hemp vegetation stages: before flowering, at flowering and at seed maturation);
(iv) Main cannabinoids in hemp inflorescence and leaf (during various growth periods) extracts;
(v) Total phenolic content (TPC) in hemp inflorescence, leaf (during various growth phases), unshelled seed and root aqueous extracts;
(vi) AA of fibre hemp root (material collected at various growth stages) extracts and EOs obtained from leaves (in different plant vegetation periods), inflorescences and unshelled seeds evaluated by the spectrophotometric DPPH^●^ scavenging assay;
(vii) AA of fibre hemp inflorescence, leaf and seed extracts by electrochemical methods, such as cyclic and square wave voltammetry;
(viii) H_2_O_2_ scavenging activity of fibre hemp roots and stems extracts.

## 2. Results

### 2.1. Soil Characteristics (Conductivity, pH and Major Elements)

The conductivity and pH of the soil of the locality where fibre hemp (*C. sativa*) was cultivated (Figure S1 in Supplementary Materials) are presented in Table 1. 

The soil textural class was a clay loam. Elemental analysis of the soil was performed by inductively coupled plasma–optical emission spectroscopy (ICP-OES), and the data are presented in Table 2.

### 2.2. Chemical Composition of Cultivated Fibre Hemp (C. sativa) EOs

The yield of fibre hemp EOs (*v*/*w*, on a dry weight basis) varied among plant organs and was highest in the flowering tops (0.33%), followed by the leaves collected before blooming in June (0.21%), the leaves gathered during the hemp flowering stage in August (0.20%) and the leaves grown at the seed maturing stage in September (0.19%). The yields of EOs slightly varied depending on the year of collection (2018–2021).

Gas chromatography (GC) equipped with FID and GC/MS (respectively, for quantitative and qualitative purposes) were applied for the chemical analysis of the hemp EOs obtained from flowers, leaves (at various plant growth stages) and stems. Principal compositional data are presented in Table 3. In total, around 50 compounds were identified in the EOs, comprising up to 98.8%.

### 2.3. Chemical Composition of VOCs in Cultivated Fibre Hemp (C. sativa) Extracts

In order to verify the bioactivity of hemp extracts, it is necessary to know the full chemical compositions (including volatiles), and not only the major bioactive constituents, such as cannabinoids, flavonoids and phenolic acids. In many cases, the synergistic effects of different compounds could play a significant role in total bioactivity. For this reason, the VOCs determined by CG/FID and GC/MS in methanolic hemp extracts (prepared according to the method described in Section 5.4.1) are presented in Table 4 and Table 5.

### 2.4. Main Cannabinoids in Fibre Hemp (C. sativa) Extracts 

In total, up to 20 compounds were identified tentatively in hemp leaf and inflorescence extracts prepared according to the method described in Section 5.4.2. The main phenolic acids and flavonoids determined in the extracts were: chlorogenic and caffeic acids, catechin, epicatechin, rutin, naringenin, quercetin-3-glucoside, apigenin-7-glucoside, apigenin, cannaflavin B and lignanamides cannabisin A, B and C. The main cannabinoids are presented in Table 6. All constituents were detected by DAD and TOF in positive or negative ionization modes. Some compounds provided *m*/*z* ions by both (positive and negative) ionizations.

### 2.5. TPC in Fibre Hemp (C. sativa) Extracts 

The TPC was determined by the Folin–Ciocalteu method [72] in various hemp extracts prepared according to the method described in Section 5.4.3 and are presented in Table 7. The highest TPC (mg/L of gallic acid equivalent (GAE)) values were identified in the aqueous extracts of hemp flowers (an average mean of 924.7 mg/L, GAE) and leaves during the flowering stage (an average mean of 922.2 mg/L, GAE). The lowest TPC (an average mean of 187.9 mg/L, GAE) was found in unshelled hemp seed extracts. The TPC values varied from 125.5 to 321.0 mg/L (GAE) in root extracts.

## 3. Antioxidant Activity (AA) of Fibre Hemp (*C. sativa*) Extracts

### 3.1. AA of Fibre Hemp Root (Material Collected at Various Growing Stages) Extracts and Inflorescence, Leaf and Unshelled Seed EOs Tested by Spectrophotometric DPPH^●^ Scavenging

The hemp extracts were prepared according to the method described in Section 5.4.4. and EOs were identified by the spectroscopic method described in the literature [62]; the obtained data are presented in Table 8. The highest AA was evaluated for hemp leaf Eos, with values ranging from 15.034 ± 0.408 to 35.036 ± 0.355 (mmol/L, TROLOX equivalent) before blooming in June and at the blooming stage in August, respectively (Table 8). In the case of root extracts, the highest AA was exhibited by root extracts at the seed maturation stage in September (1.556 mmol/L, TROLOX). 

### 3.2. AA of Fibre Hemp (C. sativa) Inflorescence, Leaf and Seed Extracts Determined by Electrochemical Methods (Cyclic and Square Wave Voltammetry)

Electrochemical techniques, including cyclic and square wave voltammetry (differing in modes of potential application to the working electrode), are widely used to obtain information about redox-active substances in solutions [73]. A metal or carbon-based working electrode is immersed in the sample and its potential is scanned in the positive direction. During the forward scan, the potential of the working electrode gradually becomes more positive, increasing the oxidizing power of the electrode. As soon as the potential of the electrode reaches the oxidation potential of the electroactive sample constituent, the oxidation of the compound occurs: the lower the potential of oxidation, the more powerful the reducing, i.e., antioxidant, properties of the compound. The oxidation (anodic) peak potential value (E_pa_) depends on the chemical structure of the electroactive substance, electrode material, pH value and composition of the solution. The magnitude of the oxidation (anodic) peak current (I_pa_) at E_pa_ is related to the concentration of the electroactive compound. During the reverse scan, reduction currents are registered. The presence of reduction (cathodic) peaks I_pc_ at reduction (cathodic) potentials E_pc_ in the reverse scan provides information about the reversibility of the redox reaction of the oxidized compounds generated in the forward scan. The E_pa_ value was suggested as a criterion for AA: compounds with oxidation potential values E_pa_ < 0.45 V were considered as antioxidants [65].

**Figure 1:**
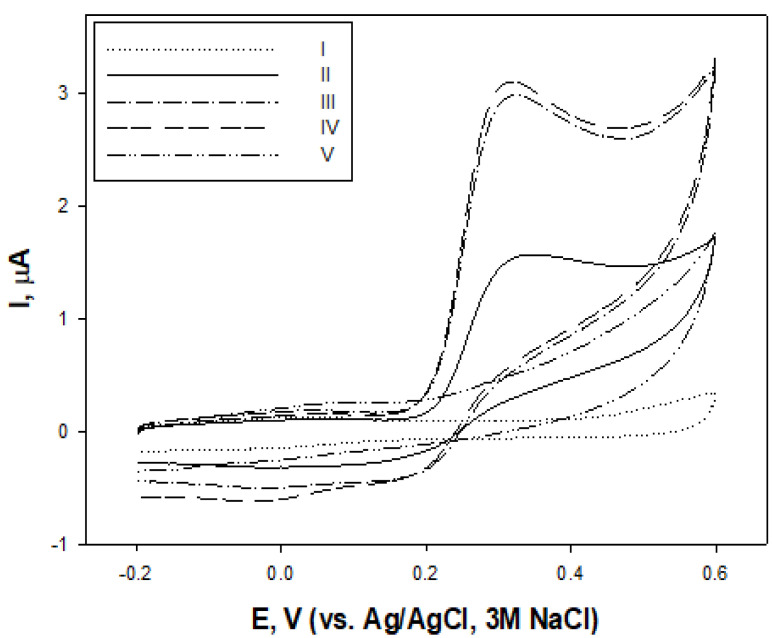
Cyclic voltammograms of carbon paste electrodes: I—in phosphate buffer at pH 7.3, II—fibre hemp (*C. sativa*) leaf (before flowering) extract, III—extract of inflorescences, IV—extract of leaves at the flowering stage and V—seed extract; potential scan rate 100 mV/s.

The cyclic voltammograms of the carbon paste electrode in *C. sativa* extracts showed the presence of anodic currents at potentials above 0.2 V (Figure 1). The similar voltammetric profiles of the flowering top and both leaf extracts suggested the presence of the same electroactive substance, with E_pa_ around 0.28 V. The increase in the currents at the potential region above 0.6 V was due to the presence of compounds with relatively high oxidation potentials. The low cathodic currents on the reverse potential scan suggested that possibly the majority of the electroactive material was oxidized irreversibly. 

**Figure 2:**
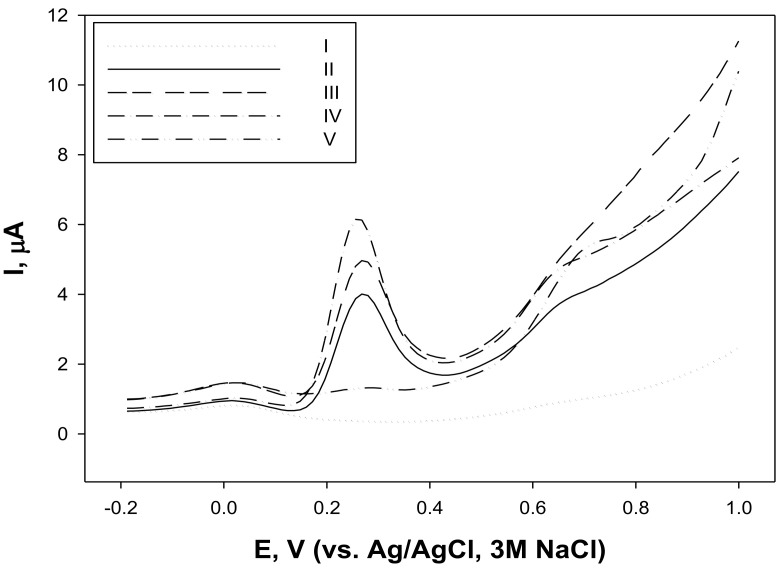
Square wave voltammograms of carbon paste electrode: I—in phosphate buffer at pH 7.3, II—fibre hemp (*C. sativa*) leaf (before flowering) extract, III—extract of inflorescences, IV—extract of leaves at the flowering stage and V—seed extract; step potential 4 mV, amplitude 50 mV and frequency 25 Hz.

Square wave voltammetry allows better resolution between species with similar redox potentials [74]. The voltammetric profiles of *C. sativa* inflorescences and both leaf extracts, again, were similar (Figure 1, traces II–IV), except for the seed extract (Figure 2, trace V). The presence of an easily oxidizable compound (E_pa_ at about 0.26 V) suggests the possible AA of hemp flowering tops and both leaf extracts.

**Figure 3:**
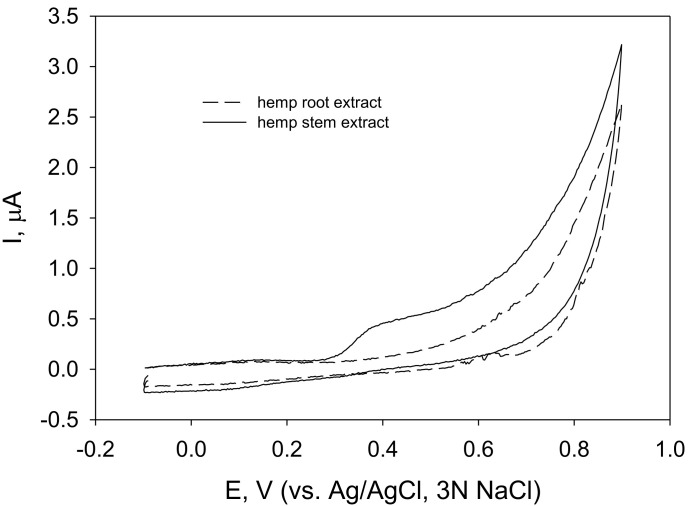
Cyclic voltammograms of carbon paste electrodes in fibre hemp (*C. sativa*) stem and root extracts (as indicated), solution pH 7.3 and potential scan rate 100 mV/s.

The cyclic voltammograms of hemp stem and root extracts (Figure 3) revealed that easily oxidizable compounds were present only in stem extracts.

### 3.3. H_2_O_2_ Scavenging Activity of Fibre Hemp (C. sativa) Root and Stem Extracts

**Figure 4:**
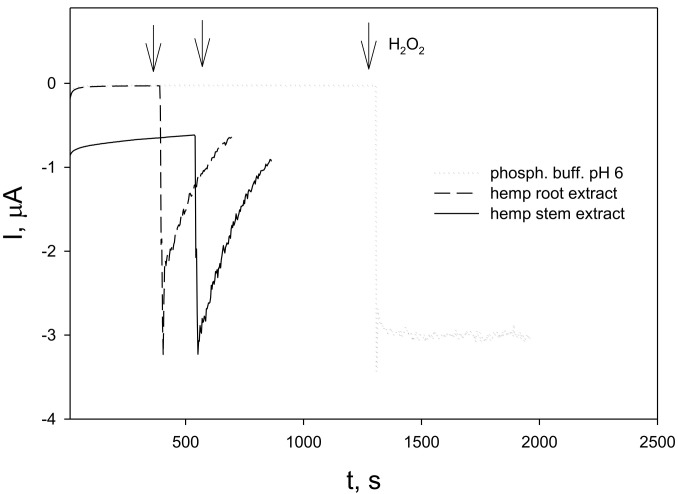
Responses of GC/PB in phosphate buffer (pH 6) fibre hemp (*C. sativa*) root and stem extracts to the addition of H_2_O_2_, operating potential 0.0 V. Arrows indicate the moments of H_2_O_2_ addition.

The electrocatalytic properties of Prussian Blue (PB) allow the reduction of hydrogen peroxide at potentials around 0.0 V, thus excluding the influence of other electroactive species [75]. The Prussian Blue-modified electrode (GC/PB) was held at a constant potential of 0.0 V until a steady state of the background current was achieved. After the injection of hydrogen peroxide into the phosphate buffer, a steady cathodic current related to hydrogen peroxide reduction at GC/PB was registered (Figure 4, dotted line). When hydrogen peroxide was injected into hemp root (Figure 4, dashed line) or stem (Figure 4, solid line) extracts, the hydrogen peroxide-induced reduction currents immediately started to decrease, indicating the disappearance of the electroactive substance, i. e., the hydrogen peroxide was scavenged by the extracts.

## 4. Discussion

Few studies have been conducted on the extracts and their biological properties of fibre hemp cultivated in Northern Europe [41,43], despite the fact that Lithuania is among the top ten producers of industrial hemp in Europe. The soil of the growth habitat (where fibre hemp was cultivated, Figure S1 in the Supplementary Materials) was investigated and characterized by some main parameters. The soil pH values ranged from 5.14 to 6.41, and the conductivity ranged from 79.14 to 143.13 (µS/cm) (Table 1). Hemp does not grow well in acidic soil; the most suitable soil for hemp cultivation should have a pH range between 5.8 and 7.7 [12,13,58,75,76,77]. Additionally, the impact of the main agronomic traits (including soil pH) was evaluated, not only on hemp biomass production, but also on the EO yield and composition [58]. To the best of our knowledge, the impact of soil acidity on the synthesis of secondary metabolites in hemp has not yet been well clarified. 

Based on the soil cover map of the Lithuanian national atlas (approved new FAO Lithuanian soil classification (LTDK-99)), the topsoil in this area was assigned to a clay loam type according to the soil textural classes [78]. It is known that heavy clay soil and sandy soil are unsuitable for hemp cultivation. The elemental composition of the soil was typical (Table 2), and the concentrations of hazardous and heavy metals were below the limitary values according to the Lithuanian health regulations (V-114 HN60:2004) [79]. Hemp needs a large amount of potassium (K) [13]. K and P (phosphorus) play a vital role in hemp fertility [12,13,58,75,76,77]; additionally, P imparts vigour and resistance against pests [6]. Some minor contents of the hazardous elements Cr (up to 2.7 ± 0.3 mg/kg) and Pb (up to 9.1 ± 1.8 mg/kg) could be explained by the human activity in this area, which is close to the city. On the other hand, hemp, being a fast-growing crop, can absorb more unwanted and harmful contaminants and toxic metals from the air, soil and water in a shorter period. 

The main bioactive compounds synthesized in fibre hemp are terpenoids, flavonoids, phenolic acids and cannabinoids. The highest contents of cannabinoids and terpenoids are found in the glandular trichomes on cannabis bracts. The highest density of glandular hairs is found on the bract surrounding each female cannabis flower and the subtending leaflets of the female inflorescence [80,81].

The amounts of EOs varied from 0.19 to 0.33% in leaves collected in September and flowering tops, respectively. The sesquiterpenoid fraction was predominant in inflorescence, and the following in leaf EOs. Monoterpenes (31.38%) dominated stem oils. Caryophyllene derivatives (*β*-caryophyllene and its oxide, caryophyla-4(12),8(13)-dien-5-α/*β*-ol and 14-hydroxy-cis-caryophyllene) were found to be the major faction in EOs of hemp flowers (46.88%) and leaves (≤68.40%) (Table 3). *Β*-Caryophyllene was found to be the predominant compound in the inflorescence (39.81%) and leaf (30.37–46.64%) EOs; and as the second (12.55%) in stem oils. *A*-Pinene (20.25%) was the major constituent of stem oils. This monoterpene was found to be the second in inflorescence oils (12.12%). *A*-Humulene was the second or third compound in the flower and leaf oils (≤11.48%). Humulene epoxide II with a quantity of 5.86% was determined to be the third main compound in the leaf (collected in June) oil. *Allo*-Aromadendrene was found to be the third major constituent (5.31%) in the stem oil. The compounds listed above were identified as principal constituents in the previous studies on industrial hemp EOs [16,28,29,30,31,32,33,34,37,38,39,49,50,51,52,56,57,58,60]. It is known that some cannabinoid compounds can be hydro-distilled together with terpene constituents in EOs. Appreciable amounts of cannabidiol (CBD, up to 4.05%) were obtained during the hydro-distillation procedure from the inflorescences, while the quantity of CBD was very low in the stem oils (Table 3). 

Among the VOCs determined in different methanolic hemp extracts, CBD dominated in all samples: from 26.09 to 64.56% in the unshelled seed and inflorescence extracts, respectively (Table 4). Appreciable quantities of 2-monolinolein (syn. glyceryl linoleic acid monoester, 11.31%), phytosterol *γ*-sitosterol (8.99%) and the ester of polyunsaturated fatty acid methyl eicosatetraenoate (syn. methyl ester of arachidonic acid, 9.70%) were determined in fibre hemp seed extracts.

(Z,Z)-9-Octadecenyl ester 9-hexadecenoic acid (10.04–31.27%), triterpenoid ketone friedelan-3-one (16.39–21.49%), *γ*-sitosterol (14.03%) and 2,3-dihydrobenzofuran (syn. Coumarin, ≤17.07%) were determined as the three main constituents of fibre hemp root (during various growth stages) methanolic extracts (Table 5). Appreciable amounts of coniferyl alcohol (monoterpene alcohol, which is a precursor for the synthesis of lignin, ≤6.35%), diterpenoid phytol acetate (8.35%) and phytosterol campesterol (7.08%) were found in root extracts. Campesterol, sitosterol and stigmasterol are considered the three most typical phytosterols found in the cannabis plant [26,70]. 

Cannabinoids are produced in the sessile and stalked trichomes of *C. sativa* plants [80,81]. Trichomes are particularly abundant on the inflorescences of the plant, present in a lower number on leaves, petioles and stems, and absent on the roots and seeds. As a consequence, the latter organs do not contain cannabinoids. The CBD quantity was the highest in hemp flower pentane extracts (32.7%) (Table 6). The lowest quantity (16.31%) of cannabidiolic acid (CBDA) was found in the hemp flowering tops. The amounts of CBDA were similar (18.20–24.21%) in fibre hemp leaf extracts during all growing stages. On the contrary, the quantity of CBD ranged drastically, from 3.24% (in leaf extracts, when herbal material was collected before the flowering stage) to 32.73% (in hemp flower extracts). A similar tendency was observed for the total content of THC isomers: traces were determined in the leaf (collected before flowering) extracts and up to 22.43% in the flower extracts. The %THC + %CBN and %CBD ratio (according to this parameter, hemp is classified into different categories) [82] was always less than 1. The hemp cultivars evaluated in this research had a ∆^9^-tetrahydrocannabinol (THC) content lower than 0.3% (calculated for dry herbal material) and satisfied the European legislation requirements for industrial hemp varieties. 

The TPCs of different plant extracts, not excluding extracts of hemp, are often difficult to compare between different sources in the literature, as there are several options for presenting these results: mg/g dry weight, mg/L extract or LC_50_ values. In addition, extracts are often prepared using different solvents, which can have a rather significant influence on the values of the parameters tested. The literature reports that the most efficient extraction of the TPC is achieved using 50% ethanol [41,47], compared with methanol and water. The TPC is also influenced by the part of the plant used to produce the extracts and the stage of vegetation of the plant itself. In our study, the highest levels of these bioactive compounds were found in the aqueous extracts of the inflorescence and leaves collected at flowering (924.7 5.5 mg/L GAE and 922.2 32.6 mg/L GAE, respectively). These results are in agreement with previously published data [41]. Even though water is not a perfect solvent for TPC extraction, the trend of the phenolic compound contents depending on the plant part and vegetation stage is in full agreement with the results published by other authors [41,47]. The lowest levels of such compounds were found in the aqueous extracts of roots, regardless of the vegetation stage (from 125.458 ± 0.000 to 320.958 ± 0.001 mg/L GAE), and in the aqueous extracts of unshelled seeds (187.9 ± 3.1 mg/L GAE. It has been reported [83] that defatted seed extracts obtained using a binary water–acetone solvent mixture had a TPC of over 50 mg GAE per g extract. The TPCs found in our study were lower, possibly due to the use of water as a solvent for extraction. 

The amount of phenolic compounds in plant tissue directly correlates with its ability to bind free radicals. The higher the TPC, the higher AA that can be expected. In our study, the free radical-scavenging capacity was determined spectrophotometrically for EOs of hemp flowers, leaves (at different stages of the plant’s vegetative growth), unshelled seeds and aqueous root extracts (at different plant growth phases). The latter showed the lowest AA (Table 8), while the highest free radical-scavenging capacity was found for EOs of leaves collected at flowering (35.036 ± 0.355 mmol TROLOX equivalent). As reported previously [83], such antioxidant effectiveness of *C. sativa* EOs may be attributed primarily to the presence of (*β*)-caryophyllene and caryophyllene oxide in high concentrations. The almost twice-lower AA value determined for inflorescence EOs (16.683 ± 0.384 mmol/L TROLOX equivalent) can confirm this statement, as flower EO contains a significantly lower amount of (*β*)-caryophyllene. Despite the fact that the TPC and the AA were tested in different matrices (aqueous extracts for TPC evaluation and EOs for AA), the results showed a similar trend, depending on the vegetation stage of the plant. Similarly to the phenolic content of the extracts, the free radical-scavenging capacity of the leaf EOs determined by the DPPH assay varied in the following order: minimum before flowering, maximum during flowering and then a decrease (Table 8). The largest number of studies on the AA of hemp have been carried out with different extracts (different parts of the plant, different solvents), and only a few have been carried out on the AA of hemp Eos [33,49].

The AA of hemp Eos and extracts was investigated and compared, not only by the conventional DPPH^●^ assay, but also by the cyclic and square wave voltammetry methods. To our best knowledge, the AA of fibre hemp extracts was evaluated for the first time by electrochemical methods. The similar voltammetric profiles (Figure 2, traces II–IV) indicated that the extracts of hemp inflorescences and both leaf extracts contained the same polyphenolic compound(s), characterized by E_pa_ 0.26 V. The I_pa_ values at this potential correlated with the TPC. Polyphenols with oxidation potentials falling in the potential region of 0.2 to 0.3 V at pH 7 are possibly compounds containing a flavonoid structure with catechol or galloyl moieties (catechin, epicatechin or quercetin) or phenolic acids (chlorogenic or caffeic). Direct comparison of obtained E_pa_ values with data from the literature is rather complicated, as the conditions of experiments (electrode material, the concentration of an electroactive substance, the presence of an organic solvent, etc.) may cause peak shifts [84]. 

ROS are inevitably produced as a by-product of normal aerobic metabolism and could be injurious for cells when present in excess under stress conditions [85]. It was considered reasonable to evaluate the antioxidant properties of polyphenols and plant extracts by their capabilities to scavenge those ROS [86]. To assess the H_2_O_2_-scavenging activity of extracts of plants, the kinetic approach by monitoring the kinetics of hydrogen peroxide scavenging at Prussian Blue (PB)-modified electrodes [87] was chosen. This approach appeared to be effective when investigating the hydrogen peroxide-scavenging activity of raspberry leaf and stem extracts [88]. Contrary to the radical-scavenging activity, the polyphenol concentration was not essential for the capability of raspberry leaf and stem extracts to scavenge hydrogen peroxide. In this study, the hemp root extract that had the lowest TPC (Table 7) scavenged hydrogen peroxide as effectively as hemp stem extract. Further, cyclic voltammograms (Figure 3) revealed that the root extract did not contain easily oxidizable compounds. It is likely that both extracts prepared at room temperature contained enzymes (such as peroxidases) that were essential for the elimination of hydrogen peroxide, as observed previously [88]. 

## 5. Materials and Methods

### 5.1. Soil Analysis

The preparation of soil samples for elemental analysis, conductivity and pH measurements was performed as follows: all samples of the soil (collected from nine different parts of the field, Figure S1 in the Supplementary Materials) were dried at room temperature, then ground and sieved through a bolt (2.0–2.5 mm of perforation).

A mixture of 20 mL of sifted soil and 40 mL of deionized water was placed for 1 h in an ultrasonic bath; later, the mixture was filtered and the conductivity of aliquots was measured using a conductivity and temperature meter, AD3000 EC/TDC (Adwa, Szeged, Hungary).

The soil preparation for pH measurements was as follows: a mixture of 5 mL of soil and 25 mL of deionized water was placed for 1 h in an ultrasonic bath; then, it was stored at room temperature for 2 h and then filtered; measurements of the pH of the aliquots were performed using a pH meter, Orion 3 Star (Thermo Fisher Scientific, Waltham, MA, USA), calibrated using buffer solutions of pH 4.01, 7.00 and 10.04. 

Elemental analyses were undertaken following the inductively coupled plasma-optical emission spectroscopy (ICP-OES) method. The procedure of sample preparation was as follows: 5 g of soil and 50 mL of 1 M HCl were stirred for 30 sec, and then left for 24 h. The mixtures were filtered and analysis was performed using an Optima 700 DV spectrometer (Perkin Elmer, Waltham, MA, USA). 

### 5.2. Plant Material

Hemp seeds for cultivation were purchased from the French centralized hemp seed growers’ cooperative. *C. sativa* L. ssp. *Sativa*: 32 plants of cultivars Futura 75 and cv. Felina (up to 2.5–3.0 kg) were collected at different vegetative stages, before blooming (June and July), at the flowering stage in August and in September (seed maturing stage), from the cultivated field (Pavilnys, Vilnius, Lithuania: 54°39’45.7” N 25°22’14.6” E) in 2018–2021. The cultivation locality is depicted on the geographic information system map (Figure S1 in the Supplementary Materials). The area of the investigated habitat was up to 2 hectares. Raw material (above and below-ground plant parts) was transported immediately to the laboratory and dried at room temperature (20–25 °C) under shade conditions for 2 weeks. The leaves, inflorescences, stems, seeds and roots were separated before drying. 

### 5.3. EO Isolation from Different Parts of Fibre Hemp (C. sativa)

The Eos from fibre hemp were isolated by the hydro-distillation of the dried material (up to 100 g each) in a Clevenger-type apparatus for 2.5 h according to the European Pharmacopoeia. The ratio of plant material to water was 1:20. A yellow–grey mass with a characteristic odour was obtained. The obtained Eos were dried over anhydrous sodium sulphate, kept in closed dark vials and stored in a refrigerator; the samples were diluted with a mixture of pentane and diethyl ether (1:1) before analysis.

Pentane and sodium sulphate (produced in India) were purchased from Sigma Aldrich Co. (St. Louis, MI, USA) and diethyl ether was obtained from C. Roth GmbH + Co. (Karlsruhe, Germany). 

### 5.4. Preparation of Various Fibre Hemp (C. sativa) Extracts for Chemical Analysis

#### 5.4.1. Extraction Procedure for GC/MS Analysis of VOCs in Hemp Methanolic Extracts 

Samples of air-dried hemp inflorescences, leaves, unshelled seeds and roots were separately ground into a homogenous powder and protected from light and humidity until analysis; ca. 2 g of crushed herbal material (5 g of roots) and 20 mL (60 mL for roots) of methanol were used for extraction. The extraction procedure was performed in an ultrasonic bath at room temperature for 30 min. The mixture was filtered through a filter paper for qualitative analysis (pore size 11 µm) and by nylon syringe filters (0.22 mm). 

Methanol was purchased from Honeywell (Seelze, Hanover, Germany).

#### 5.4.2. Preparation of Hemp Extracts for HPLC-DAD-TOF Analysis 

Samples of air-dried hemp flowers and leaves were separately ground into a homogenous powder and protected from light and humidity until analysis. The method of extract preparation was as follows: ca. 1 g of crushed herbal material and 5 mL of pentane were held in an ultrasonic bath at room temperature for 30 min. The mixture was filtered through a filter paper for qualitative analysis (pore size 11 µm) and by nylon syringe filters (0.22 mm); then, the pentane was evaporated and residuals were dissolved in acetonitrile.

Acetonitrile was purchased from Honeywell (Seelze, Hanover, Germany).

#### 5.4.3. Procedure of Preparation of Hemp Extracts for TPC and Free Radical Scavenging Capacity Measurements

An amount of 2.5 g of dry crushed plant material (flowers, leaves, seeds and roots) was added to 25 mL of distilled water and extracted in an ultrasonic bath for 30 min. The mixture was filtered and then subjected to spectrophotometric analyses for TPC and free radical-scavenging capacity determination.

#### 5.4.4. Extraction Procedure for AA Tests by Electrochemical Measurements

An amount of 5 g of ground hemp inflorescence, leaf, seed, root or stem powder was placed in 75 mL of phosphate buffer at pH 7.3 (for cyclic and square wave voltammetry) or pH 6.0 (for H_2_O_2_-scavenging test) consisting of 0.05 mM KH_2_PO_4_ and 0.1 M KCl (both from Fluka, Sigma Aldrich Chemie GMbH, Steinheim, Germany). The pH value was adjusted with KOH (Fluka). Extractions were performed with ultrasound for 30 min. The extracts were filtered through a filter paper.

### 5.5. GC Analysis of Fibre Hemp (C. sativa) Eos and Extracts

#### 5.5.1. GC/FID (Flame-Ionization Detector) Analysis 

Quantitative analyses of the Eos were carried out on an HP 5890II chromatograph equipped with an FID (Hewlett Packard, Palo Alto, CA, USA) using DB-5 ((5%-phenyl)-methylpolysiloxane; 50 m × 0.32 mm × 0.25 μm) and HP-FFAP (polyethene glycol 30 m × 0.25 mm i.d., film thickness 0.25 μm) capillary columns (Agilent, J&W Scientific, Santa Clara, CA, USA). The GC oven temperature was programmed as follows: from 50 °C (isothermal for 1 min), it was increased to 160 °C (isothermal for 2 min) at a rate of 5 °C/min, then increased to 250 °C at a rate of 10 °C/min, and the final temperature was maintained for 4 min. The temperature of the injector and detector was maintained at 250 °C. The flow rate of the carrier gas (hydrogen) was 1 mL/min. At least 3 repetitions (*n* ≥ 3) per analysis were performed.

#### 5.5.2. GC-MS Analysis of Hemp EOs

Analyses were performed on a Shimadzu GC-2010 PLUS chromatograph (Shimadzu, Kyoto, Japan) interfaced w a Shimadzu GC-MS-QP2010 ULTRA mass spectrometer (Shimadzu, Kyoto, Japan) and fitted with a capillary column Rxi-5MS (Restek, Bellefonte, PA, USA), (5%-phenyl)-methylpolysiloxane 33 m × 0.25 mm i.d., film thickness of 0.25 µm).

The conditions of chromatographic separation were the same as those for GC (FID) analysis. The temperature of the injector and detector was 250 °C. The flow rate of the carrier gas (helium) was 1 mL/min, split 1:20. At least 2 replicates (*n* ≥ 2) per analysis were performed. The temperature of the ion source was 220 °C. Mass spectra in the electron mode were generated at 70 eV, with 0.97 scans/second and a mass range of 33–400 *m*/*z*. 

#### 5.5.3. GC-MS Analysis of Hemp Methanolic Extracts

The same Shimadzu chromatograph was used for qualitative analysis; only chromatographic separation was performed under different conditions. The GC oven temperature was programmed as follows: from 60 °C (isothermal for 4 min), it increased to 330 °C (isothermal for 10 min) at a rate of 5 °C/min. The temperature of the injector and detector was maintained at 220 °C. The flow rate of the carrier gas (helium) was 1 mL/min. At least 3 replicates (*n* ≥ 3) per analysis were performed.

#### 5.5.4. Identification of Individual Components

The percentage composition of the EOs was computed from GC peak areas without correction factors. Qualitative analysis was based on a comparison of the retention indexes on both columns (polar and non-polar), co-injection of some reference terpenoids (*α*-, *β*-pinene, *β*-caryophyllene, *α*-humulene and caryophyllene oxide), CBD and C_8_–C_28_ n-alkane series; and mass spectra with corresponding data in the literature [71] and computer mass spectra libraries (Flavour and Fragrance of Natural and Synthetic Compounds 2 (FFNSC 2), Wiley and NIST). Identification was approved when computer matching with the mass spectral libraries had probabilities above 90%. The relative proportions of the oil constituents were expressed as percentages obtained by peak area normalization, with all relative-response factors being taken as one.

### 5.6. HPLC-DAD-MS (TOF) Analysis of Fibre Hemp (C. sativa) Extracts

Extracts (prepared according to Section 5.4.2) of the inflorescences, leaves and roots were analysed by the HPLC technique using an HPLC/diode array detector (DAD)/time-of-flight (TOF) system (Agilent 1260 Infinity (Agilent Technologies, Waldbronn, Germany) and an Agilent 6224 TOF (Agilent Technologies, Santa Clara, CA, USA) system equipped with a reverse phase column ZORBAX Eclipse XDB (C18, 5 μm particle size, 150 × 4.6 mm, Agilent Technologies, Santa Clara, CA, USA). The column temperature was maintained at 35 °C. A gradient system was applied: A (deionized water, containing 0.1% of formic acid) and B (acetonitrile, containing 0.1% of formic acid). Chromatographic separation was performed at a flow rate of 0.7 mL/min in the HPLC system with the following stepwise gradient elution method: from 0 to 2 min, initial ratio of 40% (A)/60% (B); from 2 to 9 min, from the initial ratio to 30% (A)/70% (B); from 9 to 13 min, isocratic mode at 30% (A)/70% (B), from 13 to 29 min, from 30% (A)/70% (B) to 10% (A)/90% (B); and from 29 to 34 min, from 10% (A)/90% (B) to the initial ratio 40% (A)/60% (B) and the isocratic mode for 1 min at 40% (A)/60% (B). Ionization was performed by electrospray ionization interface (ESI) in positive and negative modes. A sample volume from 10 to 15 μL was injected by an auto-sampler. 

The MS (TOF) acquisition parameters were as follows: mass range 100–1700 *m*/*z*, rate 1.42 spectra/s and time 704.2 ms/spectrum. The ionization source conditions were: drying gas temperature 300 °C, drying gas flow rate 3 L/min, nebulizer 15 psig, fragmentor voltage 125 V and skimmer 65 V. To assure the mass accuracy of the recorded data, continuous internal calibration was performed with reference masses *m*/*z*: 121.0509, 149.0233, 322.0481, 922.0098, 1221.9906 and 1521.9715 (as per instrument standards, ref. nebulizer 5 psig). 

Acetonitrile was purchased from Honeywell (Seelze, Hanover, Germany) and formic acid was purchased from Sigma Aldrich Co. (St. Louis, MI, USA).

### 5.7. TPC in Hemp (C. sativa) Extracts

The TPC was determined in hemp extracts using the Folin–Ciocalteu assay [72].

Amounts of 20 μL of hemp inflorescence, leaf, seed and root extract and 1580 μL of distilled water were added to 100 μL of the Folin–Ciocalteu reagent and 300 μL of Na_2_CO_3_ (20% *w*/*v*). The mixture was left under darkness at room temperature for 2 h. The absorbance at a 765 nm wavelength was measured using a spectrophotometer (UV/Vis Lambda 25, Perkin Elmer, Buckinghamshire, UK). The results are expressed in mg/L GAE. The calibration curve used for calculations (Figure S2 in Supplementary Materials) was obtained using different concentrations of gallic acid: 0,00, 50, 100, 150, 250 and 500 mg/L. All measurements were performed in triplicate.

### 5.8. Spectrophotometric DPPH Radical Scavenging Assay

The DPPH^●^ radical scavenging method reported in the literature [62] was modified as described in our previous study [88]. A 6 × 10^−5^ M stock solution of DPPH^●^ was obtained by dissolving 2,2-diphenyl-1-picrylhydrazyl in methanol. The working solution was prepared by diluting the stock solution with methanol to obtain an absorbance value of 0.730 ± 0.02 at 515 nm. The hemp extracts for analysis were diluted 1:50 with a mixture of methanol and water (80:20); 0.1 mL of the prepared sample was allowed to react with 3.9 mL of working DPPH^●^ solution under darkness for 30 min. Thereafter, the absorbance of the reacted mixture was measured. The results are expressed in mmol/L TROLOX equivalent. Five milligrams of TROLOX (±)-6-hydroxy-2,5,7,8-tetra-methylchromane-2-carboxylic acid) were dissolved in a methanol and water solution (70:30) and diluted to 100 mL. Five different concentrations from this solution were prepared (200, 100, 50, 25 and 12.5 mmol/L). An amount of 0.1 mL of each TROLOX solution was allowed to react with 3.9 mL of the working solution of DPPH^●^. The absorbance values were measured at 515 nm after 30 min. The absorbance was measured using a spectrophotometer (UV/Vis Lambda 25, Perkin Elmer, Buckinghamshire, UK). Linear calibration curves (Figure S3 in Supplementary Materials) were obtained, and their parameters were used for further calculations of antioxidant capacity. All measurements were performed in triplicate.

### 5.9. Electrochemical (Cyclic and Square Wave Voltammetry) Analysis

Amperometric measurements were performed with a BAS-Epsilon Bioanalytical system (West Lafayette, IN, USA). The conventional three-electrode cell contained a carbon paste electrode as a working electrode, platinum as an auxiliary electrode and Ag/AgCl, 3 N NaCl as a reference electrode. The carbon paste electrode was prepared by thoroughly mixing 200 mg of graphite powder with 100 µL of paraffin oil. The paste was packed into the cavity of a homemade electrode consisting of a plastic tube (2.9 mm) and a copper wire serving as an electrode contact. The surface of the electrode was thereafter smoothed on white paper.

Cyclic and square wave voltammetry analyses at the carbon paste electrode were performed for the hemp leaf, flowering top and seed extracts in phosphate buffer (0.025 M KH_2_PO_4_ and 0.1 M KCl) at pH 7.3. The pH value was adjusted with KOH. Cyclic voltammograms were recorded in the potential region −0.2 to 1.0 V at a potential scan rate of 100 mV/s. Square wave voltammograms were recorded under the following conditions: step potential of 4 mV, amplitude of 50 mV and frequency of 25 Hz

### 5.10. Hydrogen Peroxide Scavenging Test 

The tests were performed according to the method presented in our previous research [88]. Prior to the electrodeposition of PB, the glassy carbon electrode was polished with Al_2_O_3_ to a mirror finish and sonicated in water for 2 min. PB was electrodeposited from a solution containing 2.5 mM of FeCl_3_, 2.5 mM of K_3_[Fe(CN)_6_], 0.1 M of KCl (all from Fluka) and 0.1 M of HCl (Reakhim, Moscow, Russia) by applying 400 mV for 40 s. Thereafter, the electrode was transferred to a solution containing 0.1M KCl and 0.1 M HCl and cycled between 350 mV and −25 mV 25 times (potential scan rate 25 mV/s).

To assess the hydrogen peroxide scavenging activity, GC/PB was held in phosphate buffer at pH 6 or hemp root and leaf extracts at 0.0 V until a steady state of the background current was achieved. Hydrogen peroxide solution was then added to a final concentration of 0.15 mM.

## 6. Statistical Analysis

The obtained results were statistically processed by calculating the Pearson correlation coefficient (*r*); the results were expressed as mean values, range intervals and standard deviation (SD) values, using XLSTAT (trial version, Addinsoft 2014, Paris, France). 

## 7. Conclusions

The major parameters identified in the topsoil of the field (where fibre hemp was cultivated) contribute to the still unclear agronomic guidance and fertilization recommendations for hemp cultivation. 

The amount and the chemical composition of hemp EOs and various extracts depended on the conditions of the extract preparation technique, on the plant organ and the time of collection of the plants. It was found that stem EOs were the most distinguishable by their quantitative composition. Hemp flower and leaf EOs were characterized by significant amounts of caryophyllene derivatives. The roots were determined to be a valuable source of bioactive compounds, as well as the above-ground part of hemp (flowering tops and leaves). The content of principal compounds in roots (friedelan-3-one, octadecenyl ester of hexadecenoic acid, coumarin and *γ*-sitosterol) varied strongly according to the hemp growing stage. The high contents of CBD (≤32.73%) and CBDA (≤24.21%) were characteristic of the investigated hemp extracts. Studies on the TPC and free radical-scavenging capacity of the hemp extracts and EOs showed that the extracts of leaves and flowers and their EOs (during the flowering period) were the richest in bioactive compounds and exhibited the highest antioxidant properties. Although the root extracts did not show high TPC or AA, they contained valuable compounds known for other their bioactivities. The electrochemical assays revealed the presence of easily oxidizable compounds (antioxidants) with characteristic oxidation potentials E_pa_ of 0.26 V (vs. Ag/AgCl, 3 N NaCl), as determined by square wave voltammetry. The I_pa_ values correlated with TPC. The AA of fibre hemp extracts was evaluated for the first time by electrochemical (cyclic and square wave voltammetry) methods. The TPC did not influence the hydrogen peroxide-scavenging activity. 

This paper adds to the limited number of studies on the AA of fibre hemp extracts and EOs in particular. Hemp can be cultivated not only for its fibre, but also as a source of bioactive compounds. This will provide new application opportunities in various fields of science and industry due to the medical, nutritional and nutraceutical benefits of industrial hemp. Additionally, we need to mention that fibre hemp can be cultivated without restrictions to farmers. 

In order to obtain much more information about the bioactivity of different extracts and/or new preparation techniques from various fibre hemp plant organs (especially roots), the research on the topic will be continued. Future investigations should be focused on trace cannabinoids (other than THC, CBN, CBD and CBDA), the exploration of their pharmacological properties and the development of hemp-based products. Due to hemp’s ability to suppress weed growth, research on the allelopathic properties of fibre hemp extracts would be promising and important for agronomy and agriculture. 

## Figures and Tables

**Table 1:** Conductivity (µS/cm) and pH values of soil (*n* = 3, average mean, SD—standard deviation) at nine different sampling sites (I–IX) of the fibre hemp (*C. sativa)* growing location.

Sampling Site	Conductivity, µS/cm, SD	pH Value, SD
I	79.14 (2.23)	5.78 (0.31)
II	143.13 (3.56)	6.04 (0.21)
III	119.22 (2.23)	6.09 (0.05)
IV	114.09 (4.2)	6.16 (0.14)
V	131.78 (3.24)	6.41 (0.13)
VI	89.55 (4.07)	5.34 (0.14)
VII	100.55 (3.22)	5.14 (0.31)
VIII	97.49 (1.20)	5.83 (0.22)
IX	103.45 (5.45)	5.45 (0.12)

**Table 2:** Main elements * (mg/kg, *n* = 3, average mean, SD—standard deviation) in the soil (nine sampling sites, I–IX, Figure S1) of the fibre hemp (*C. sativa*) growing location.

						mg/kg							
Sampling Sites	Ca	Mg	K	Na	Al	Mn	Cu	Cd	Cr	Ni	Pb	Zn	P
λ, nm	317.93	285.21	766.49	589.59	396.15	257.61	327.39	228.80	267.72	231.60	220.35	213.86	231.67
I	1057.0	142.2	111.5	58.7	1626.6	324.7	7.1	0.0	0.0	0.0	5.0	15.3	365.2
SD	27.0	2.1	3.4	1.0	31.1	4.4	1.2	0.0	0.0	0.0	1.3	0.6	26.9
II	928.6	142.1	100.8	71.6	1592.3	284.0	4.3	0.0	0.1	0.0	2.9	13.8	297.2
SD	11.7	1.7	3.3	3.4	10.0	7.3	0.4	0.0	0.0	0.0	0.8	1.3	11.6
III	1071.0	136.9	85.9	58.4	1728.6	333.3	6.0	0.0	0.1	0.0	3.6	14.2	373.6
SD	29.5	2.4	6.0	2.2	5.5	1.0	1.0	0.0	0.1	0.0	1.9	3.7	15.6
IV	1479.0	179.0	77.0	83.6	1831.3	421.9	5.9	0.0	0.0	0.0	6.0	14.8	414.0
SD	47.1	4.5	1.5	3.1	50.1	9.0	1.4	0.0	0.0	0.0	0.2	0.5	17.7
V	2343.0	211.1	58.0	62.1	1664.6	376.2	14.3	0.0	0.2	0.0	3.7	20.9	556.7
SD	62.8	9.2	4.1	2.8	61.6	9.3	1.3	0.0	0.1	0.0	1.3	1.8	6.1
VI	1094.0	217.8	132.5	82.6	1694.0	299.1	12.9	0.0	1.9	0.0	5.1	11.5	369.6
SD	48.2	4.9	8.6	4.2	20.2	2.4	2.6	0.0	0.1	0.0	1.1	2.3	14.6
VII	1525.0	277.8	156.6	73.4	2116.3	390.4	9.4	0.0	2.2	0.0	7.3	16.1	489.9
SD	75.3	3.6	8.2	2.9	39.5	5.0	1.6	0.0	0.4	0.0	1.9	2.0	74.9
VIII	1501.6	262.7	77.8	60.3	2144.6	402.2	8.5	0.1	2.2	0.0	9.1	16.3	513.6
SD	34.7	4.2	7.9	3.1	84.8	9.0	0.6	0.0	0.2	0.0	1.8	1.7	32.6
IX	1439.0	249.3	37.2	42.3	2261.3	436.1	7.8	0.0	2.7	0.0	7.5	21.3	516.2
SD	23.1	2.9	6.3	3.7	71.1	7.3	0.7	0.0	0.3	0.0	0.5	1.8	18.1

* Ca—calcium, Mg—magnesium, K—potassium, Na—sodium, Al—aluminium, Mn—manganese, Cu—copper, Cd—cadmium, Cr—chromium, Ni—nickel, Pb—lead, Zn—zinc and P—phosphorus.

**Table 3:** Main chemical composition (≥3.0%) of inflorescence, leaf collected in June (before flowering), August (during flowering stage) and September (seeds maturing stage)) and stem EOs of fibre hemp (*C. sativa*) (*n* = 3, average mean ± SD (standard deviation), plants collected from nine sites in the investigated area.

				%			
Compound ^a^	^b^ RI_Lit_	^c^ RI_Exp_	Flowers	Leaves (in June)	Leaves (in August)	Leaves (in September)	Stems
*α*-Pinene *	939	938	12.12 ± 5.81	2.94 ± 0.28	1.73 ± 0.84	4.76 ± 1.60	20.25 ± 3.38
*β*-Pinene *	980	984	1.89 ± 1.00	0.70 ± 0.06	5.31 ± 3.30	1.59 ± 0.97	4.82 ± 1.51
*β*-Myrcene	991	990	5.49 ± 3.40	0.38 ± 0.30	0.85 ± 0.25	0.95 ± 0.05	4.31 ± 0.04
*β*-Caryophyllene *	1419	1415	39.81 ± 7.31	30.37± 4.25	46.64 ± 4.25	46.54 ± 3.67	12.55 ± 2.04
*α*-*trans*-Bergamotene	1436	1439	3.14 ± 0.04	2.85 ± 0.41	1.00 ± 0.03	3.58 ± 0.05	0.01 ± 0.01
*β*-*trans*-Farnesene	1443	1445	4.76 ± 0.42	2.39 ± 0.95	0.67 ± 0.33	4.55± 0.21	
*α*-Humulene *	1455	1461	11.48 ± 1.82	10.78 ± 1.71	10.76 ± 5.42	10.40 ± 1.67	3.56 ± 1.01
*allo*-Aromadendrene	1461	1465	0.64 ± 0.58	1.25 ± 0.58	0.94 ± 0.50	0.57 ± 0.33	5.31 ± 2.07
*α*-Selinene	1498	1503	1.37± 0.38	2.68 ± 0.26	3.26 ± 0.77	4.07 ± 4.79	0.02 ± 0.01
Caryophyllene oxide *	1580	1586	4.13 ± 0.32	14.53 ± 2.96	10.24 ± 1.36	4.64 ± 2.66	3.14 ± 0.59
Humulene epoxide II	1606	1615	1.23 ± 0.14	5.86 ± 0.72	4.23 ± 1.25	3.23 ± 3.09	1.64 ± 1.54
Caryophyla-4(12),8(13)-dien-5-*α*-ol	1640	1639	0.71± 0.14	2.50 ± 0.76	3.70 ± 0.70	2.01 ± 0.94	0.04 ± 0.02
Caryophyla-4(12),8(13)-dien-5-*β*-ol	1640	1641	0.75± 0.08	3.08 ± 0.52	4.70 ± 0.20	2.75 ± 1.02	0.05 ± 0.01
*allo*-Himachalol	1662	1660	0.01 ± 0.01	3.50 ± 0.45	0.01 ± 0.01	2.54 ± 0.42	0.21 ± 0.11
14-hydroxy- *cis*-Caryophyllene	1667	1668	1.48 ± 0.01	3.54 ± 0.38	3.12 ± 1.02	2.85 ± 0.02	0.01 ± 0.01
Cannabidiol (CBD) *	-	2383	4.05 ± 0.56	0.41 ± 0.10	3.05 ± 0.05	3.08 ± 0.02	0.04 ± 0.00
Caryophyllene derivatives (average sum)			46.88	54.02	68.40	58.79	15.79

^a^ Constituents are listed in order of their elution from a non-polar DB-5 (which is identical to a Rxi-5MS) column; compounds were identified by their mass spectra and retention indices on both (polar HP-FFAP and nonpolar Rxi-5MS) columns; ^b^ RI_Lit_: Kovat’s indices for the nonpolar column DB-5 taken from the literature [71]; ^c^ RI_Exp_: retention indices determined experimentally on the nonpolar column Rxi-5MS. * Additional identification with reference compounds.

**Table 4:** Chemical composition (%) of VOCs in methanolic inflorescence, leaf (collected in August, during flowering stage) and unshelled seed extracts of cultivated fibre hemp (*C. sativa*) (*n* = 3, average mean ± SD, plants collected from nine sites in the investigated area).

		%		
Compound ^a^	^b^ RI_Exp_	Flowers	Leaves (in August)	Unshelled Seeds
Heptanal	908			1.75 ± 0.25
*β*-Caryophyllene	1415	4.67 ± 1.01	3.77 ± 2.25	
*β*-*trans*-Farnesene	1445	0.86 ± 0.22	0.58 ± 0.33	
*α*-Humulene	1461	1.18 ± 0.80	0.83 ± 0.44	
Caryophyllene oxide	1586		1.14 ± 0.66	
Humulene epoxide II	1615		0.53 ± 0.25	
14-hydroxy-*cis*-Caryophyllene	1666	0.69 ± 0.41	1.10 ± 0.83	
*epi*-*α*-Bisabolol	1686		0.77 ± 0.53	
Neophytadiene	1840		1.01 ± 0.80	0.93 ± 0.33
Heptadecanoic acid	2084			2.15 ± 1.02
9,12-Octadecadienoic acid methyl ester	2102			0.77 ± 0.05
Methyl linoleate	2109			0.50 ± 0.21
Phytol	2117	1.91 ± 0.83	2.28 ± 0.32	1.92 ± 1.00
Oleic acid	2140		0.94 ± 0.58	
Canabichromene	2368	0.50 ± 0.32	0.46 ± 0.28	
Cannabidiol	2383	64.56 ± 2.58	48.41 ± 4.05	26.09 ± 2.75
3-Cyclopentylpropionic acid, 2-dimethylaminoethyl ester	2423			3.12 ± 1.01
Dronabinol	2470	1.98 ± 0.41	2.21 ± 0.98	0.54 ± 0.41
Hexadecanoic acid, 2-hydroxy-1-(hydroxymethyl) ethyl ester	2498			3.75 ± 0.97
2-Octyl-1-dodecanol	2512	0.97 ± 0.08		
2-Monolinolein	2606			11.31 ± 1.92
(Z)-5,11,14,17-Methyl eicosatet-raenoate	2674	0.89 ± 0.28		9.70 ± 2.25
2,3-Dihydroxypropyl-octadecanoic acid	2714			1.47 ± 0.67
Cannabigerol	2748		0.36 ± 0.08	
*α*-Tocopherol	3109		0.71 ± 0.63	2.70 ± 0.15
Campesterol	3110			2.12 ± 0.33
*γ*-Sitosterol	3341	2.16 ± 0.88	1.61 ± 0.09	8.99 ± 2.71
Fucosterol	3345			0.88 ± 0.10
*β*-Amirine	3355	0.63 ± 0.28	2.71 ± 0.88	
*α*-Amirine	3376	0.60 ± 0.19	1.74 ± 0.96	
Stigmast-4-en-3-one	3458			1.06 ± 0.03

^a^ Constituents are listed in order of their elution from a non-polar DB-5 (which is identical to a Rxi-5MS) column; compounds were identified by their mass spectra and retention indices on both (polar HP-FFAP and nonpolar Rxi-5MS) columns; ^b^ RI_Exp_: retention indices determined experimentally on the nonpolar column Rxi-5MS.

**Table 5:** Major (≥3.0%) composition of VOCs in the root (root material collected at different hemp vegetation stages: before flowering, during flowering and at seed maturing phases) extracts of fibre hemp (*C. sativa*) (*n* = 3, average mean ± SD, plants collected from nine sites in the investigated area).

		%		
Compound ^a^	^b^ RI_Exp_	Roots (Plants before Flowering)	Roots (at Flowering Stage)	Roots (at Seeding Stage)
Piranone	988	2.61 ± 1.22	1.09 ± 0.93	3.07 ± 0.91
2,3-Dihydrobenzofuran	1225	17.07 ± 2.54	9.01 ± 1.04	14.19 ± 1.08
(*E*)-Coniferyl alcohol	1734	6.35 ± 0.92	1.75 ± 1.07	4.47 ± 0.78
Palmitic (hexadecanoic) acid	1974	4.64 ± 1.73	1.26 ± 0.04	0.04 ± 0.01
Campesterol	3110	1.87 ± 0.55	1.11 ± 0.44	7.08 ± 2.66
Stigmasterol	3310	1.36 ± 0.22	0.76 ± 0.33	2.98 ± 1.45
*γ*-Sitosterol	3341	6.64 ± 1.77	14.03 ± 1.08	13.99 ± 2.42
Stigmastanol	3349	3.09 ± 0.41	0.11 ± 0.04	0.01 ± 0.01
*β*-Amirine	3355	3.06 ± 0.47	2.25 ± 0.55	1.26 ± 0.42
Phytol acetate *	3488	0.21 ± 0.21	8.35 ± 1.01	0.09 ± 0.05
Friedelan-3-one	3510	21.49 ± 3.03	16.39 ± 2.82	16.96 ± 3.01
(Z,Z)- 9-Octadecenyl ester 9-hexadecenoic acid	3515	10.04 ± 0.77	31.18 ± 2.33	31.27 ± 1.77

^a^ Constituents are listed in order of their elution from a non-polar DB-5 (which is identical to a Rxi-5MS) column; compounds were identified by their mass spectra and retention indices on both (polar HP-FFAP and nonpolar Rxi-5MS) columns; ^b^ RI_Exp_: Retention indices determined experimentally on the nonpolar column Rxi-5MS. * Mass spectrum was identical to the phytol acetate spectrum; mass spectrum libraries identified the compound as phytol acetate with a probability of more than 85%, despite the fact that RI _Exp_ differed from the retention index reported in the literature [71].

**Table 6:** Content (%, *n* = 3, average mean ± SD, plants collected from nine sites in the investigated area) of main cannabinoids in fibre hemp (*C. sativa)* extracts.

		%		
Plant Organ	CBN Observed *m*/*z* [M+H]^+^ 311.43, Da	CBDA Observed *m*/*z* [M+H]^+^ 359.22, Da	CBD * Observed *m*/*z* [M+H]^+^ 315.23, Da	Total Sum of THC Isomers
Leaves before flowering	n.d.	24.11 ± 2.04	3.24 ± 0.43	0.10 ± 0.01
Flowering tops	n.d.	16.31 ± 1.85	32.73 ± 2.70	22.43 ± 2.04
Leaves in flowering stage	n.d.	18.20 ± 1.31	26.84 ± 1.61	10.53 ± 0.72
Leaves in seed maturing stage	n.d.	24.21 ± 3.02	26.54 ± 2.03	3.45 ± 0.98

* Additional identification with reference compound; n.d.—not determined, amount below detection limits; CBN—cannabinol; CBDA—cannabidiolic acid; CBD—cannabidiol; THC—tetrahydrocannabinol.

**Table 7:** TPC (mg/L of GAE, *n* = 3, average mean (SD), plants collected from nine sites in the investigated area) of hemp (*C. sativa*) aqueous extracts.

PLANT ORGAN	Leaves before Flowering	Flowering Tops	Leaves in Flowering Stage	Leaves in Seed Maturing Stage	Unshelled SEEDS	Roots
TPC, mg/L GAE	422.2 (16.6)	924.7 (5.5)	922.2 (32.6)	573.9 (31.7)	187.9 (3.1)	223.2 (10.8)

**Table 8:** AA (mmol/L TROLOX equivalent) of fibre hemp (*C. sativa*) root extracts (at different vegetative stages) and EOs obtained from leaves (at different plant vegetation stages), inflorescences and unshelled seeds evaluated by DPPH^●^ assay.

Root Extract	DPPH^●^ Scavenging Activity TROLOX (mmol/L)	Essential Oil	DPPH^●^ Scavenging Activity TROLOX (mmol/L)
Before blooming (June)	0.290 ± 0.116	Leaf (before blooming/June)	15.034 ± 0.408
Before blooming (Jully)	0.562 ± 0.166	Leaf (before blooming/Jully)	21.662 ± 0.772
Flowering stage (August)	1.023 ± 0.005	Inflorescences (August)	16.683 ± 0.384
Seed maturation stage (September)	1.556 ± 0.004	Leaf (blooming/August)	35.036 ± 0.355
		Leaf (seed maturation stage/September)	20.311 ± 0.171
		Unshelled seeds (September)	13.187 ± 0.758

## Data Availability

Not applicable.

## Supplementary Materials

The following supporting information can be downloaded at: https://www.mdpi.com/article/10.3390/molecules28134928/s1, Figure S1: Geographical indication of fibre hemp cultivated field and sampling sites (Pavilnys, Vilnius, Lithuania: 54°39′45.7″ N 25°22′14.6″ E); Figure S2: Gallic acid standard calibration curve (Folin–Ciocalteu method); Figure S3: TROLOX standard calibration curve.

## Sample Availability

Samples of plant extracts are available from the authors.
