# Supplementary material for: Phytochemical Composition and Antioxidant Activity of Various Extracts of Fibre Hemp (Cannabis sativa L.) Cultivated in Lithuania
Source: Molecules. 2023 Jun 22;28(13):4928. doi: 10.3390/molecules28134928 (PMC10343532; doi:10.3390/molecules28134928)
Supplement: Supplementary file 1 [file molecules-28-04928-s001.zip › molecules-2417998-supplementary.pdf]

Phytochemical composition and antioxidant activity of various extracts of fibre hemp (*Cannabis sativa* L.) cultivated in Lithuania

Asta Judžentienė, Rasa Garjonytė and Jurga Būdienė

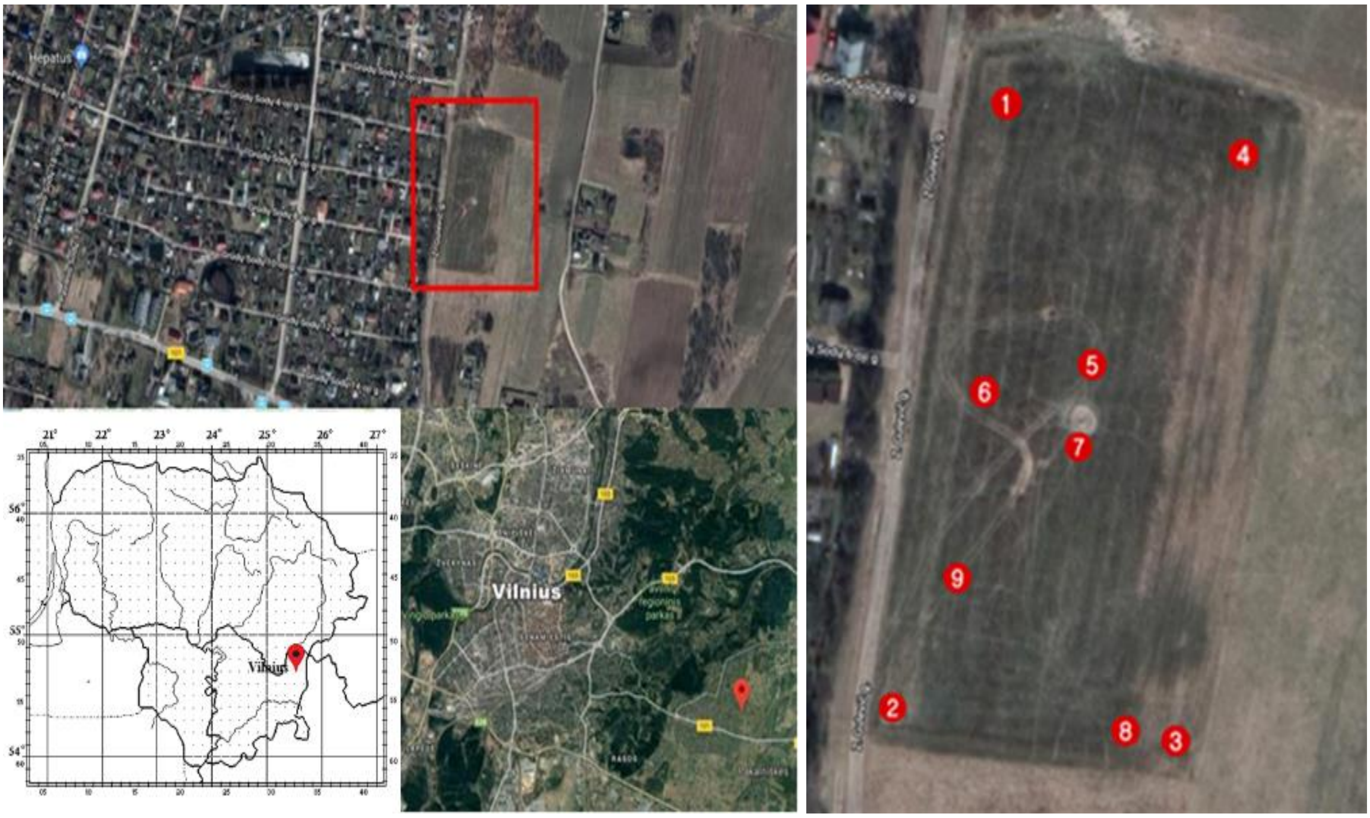

Figure S1. Geographical indication of fibre hemp cultivated field and sampling sites (Pavilnys, Vilnius, Lithuania: 54°39'45.7"N 25°22'14.6"E).

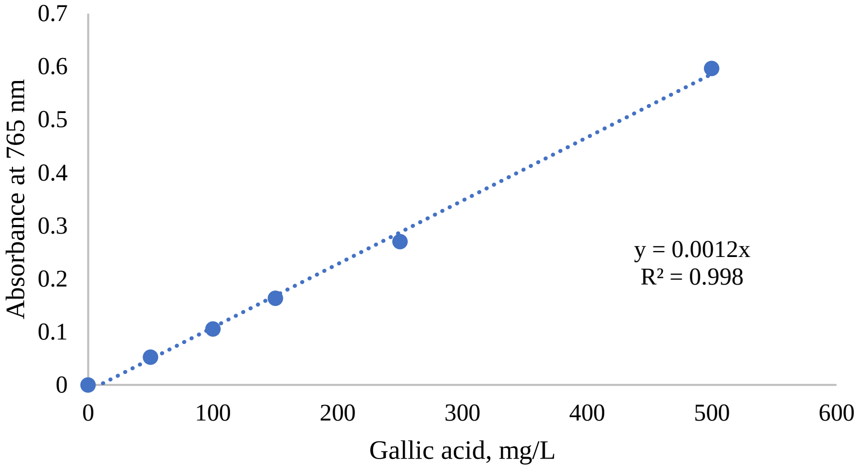

Figure S2. Gallic acid standard calibration curve (Folin–Ciocalteu method).

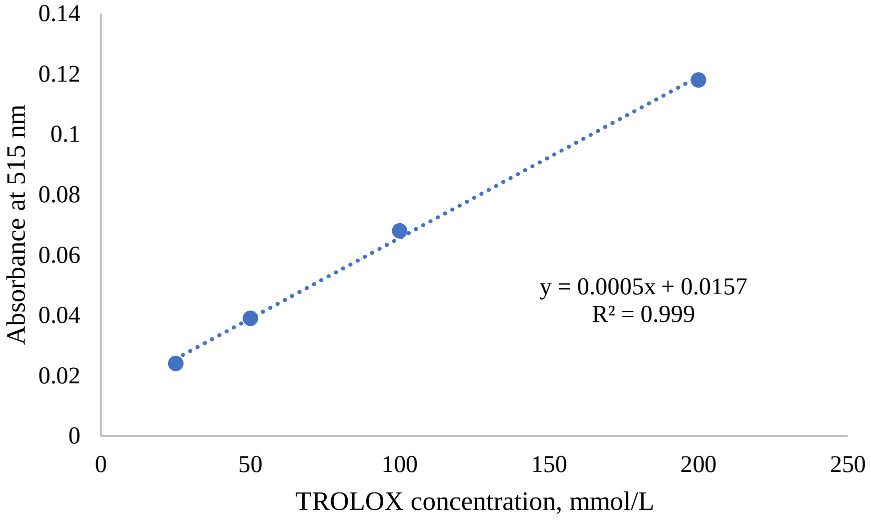

Figure S3. TROLOX standard calibration curve.
